# MicroRNA Profiling Reveals Distinct Profiles for Tissue-Derived and Cultured Endothelial Cells

**DOI:** 10.1038/s41598-017-11487-4

**Published:** 2017-09-08

**Authors:** Suvi M. Kuosmanen, Emilia Kansanen, Virve Sihvola, Anna-Liisa Levonen

**Affiliations:** 0000 0001 0726 2490 grid.9668.1 A.I.Virtanen Institute, University of Eastern Finland, Kuopio, 70211 Finland

## Abstract

Endothelial plasticity enables the cells to switch their phenotype according to the surrounding vascular microenvironment. MicroRNAs (miRNAs) are small noncoding RNAs that control endothelial plasticity. The objective of this study was to investigate the differences in miRNA profiles of tissue-derived cells and cultured endothelial cells. To this end, miRNA expression was profiled from freshly isolated tissue-derived human vascular endothelial cells and endothelial cells cultured until cellular senescence using miRNA sequencing. In addition, the data was searched for putative novel endothelial miRNAs and miRNA isoforms. The data analysis revealed a striking change in endothelial miRNA profile as the cells adapted from tissue to cell culture environment and the overall miRNA expression decreased significantly in cultured compared to tissue-derived endothelial cells. In addition to changes in mechanosensitive miRNA expression, alterations in senescence-associated and endothelial-to-mesenchymal-transition-associated miRNAs were observed in aging cells. Collectively, the data illustrates the adaptability of endothelial cell miRNA expression that mirrors prevailing cellular environment.

## Introduction

MicroRNAs (miRNAs) are approximately 22 nt long small non-coding RNAs that post-transcriptionally regulate gene expression. They elicit their regulatory function through binding their cognate mRNA transcripts to repress or activate translation or to cause mRNA turnover and degradation^1^. Currently, the central repository for miRNAs, miRBase (v21)^2^, catalogues 2588 human miRNAs, but recent studies suggest that there are many more to be found, especially those that are lineage-, tissue- and cell-specific^3^. In miRNA biology, it is notable that only a few hundred miRNAs are sufficiently expressed at any given moment to affect post-transcriptional gene regulation^4^.

Although most cellular miRNAs are scarcely expressed, their expression is often increased in pathological states resulting in a shift in the cellular miRNA profile^5^. Despite advances made in the miRNA field, currently most of the miRNA profiling studies have been executed in tissue samples. However, tissue analysis does not provide information on the distinct expression patterns of the different cell types that constitute the tissue. This limitation has led to some misconceptions in cellular miRNA expression and to studies of miRNA function in irrelevant cell types^6^. Therefore, studies on cell type-specific miRNA profiles are crucial for enhancing our understanding of miRNA biology.

In blood vessels, a single layer of endothelial cells maintains an interface between blood and tissues, surrounded by adjacent cells and extracellular matrix that influence their phenotype. For example, the composition and stiffness of the extracellular matrix is critical for endothelial cell survival and stability of the endothelial barrier. In addition to extracellular matrix, other cell types directly or indirectly interact with the cells. Furthermore, chemical stimuli, such as varying oxygen levels, paracrine signals and plasma constituents, as well as mechanical forces, such as shear stress and cyclic stress from ventilation, affect endothelial function^7^. In tissue environment, the plasticity of endothelial cells allows them to switch their phenotype to match the surrounding requirements, for example from quiescence to growth to accomplish vascularization of hypoxic areas^8^. Upon isolation, however, endothelial cells undergo a major change in their extracellular environment to adapt to new one.

In tissue environment, endothelial cells are quiescent dividing only in response to injuries or specific signals^9^. Extraction from tissue environment and transfer to cell cultures activates cells and induces proliferation, which eventually leads to cellular senescence, as the cells reach their replicative limit. Harmful stress stimuli, such as oxidative stress or extensive cell divisions can lead to premature senescence and biologically older cells than their chronological age suggests^10^. Aging has been shown to affect endothelial function strongly by predisposing to endothelial dysfunction, and thus promoting the development of aging-related disorders^11^.

In this study, we have explored the changes in endothelial miRNA profile from tissue-derived to cultured cells and from young to old cells using miRNA sequencing (miRNA-seq). Furthermore, we have extracted putative novel endothelial miRNAs and miRNA isoforms (isomiRs) from the data. The data analysis revealed a significant change in endothelial miRNA profile as the cells adapted from tissue to cell culture environment. In addition to changes in mechanosensitive miRNA expression, miRNAs associated with senescence inhibition and induction were downregulated and upregulated, respectively, in aging cells. Furthermore, a shift towards mesenchymal miRNA profile was observed in the aging endothelial cells. Collectively, the data illustrates the plasticity of endothelial cells, and elucidates the fluid nature of the “cell-specific” miRNA profiles, clearly emphasising that the cellular miRNA profile depends not only on the cell type and developmental stage but also on the prevailing environmental cues affecting the cells.

## Results

### Endothelial miRNA Profile: from Flow to Static

**Figure 1:**
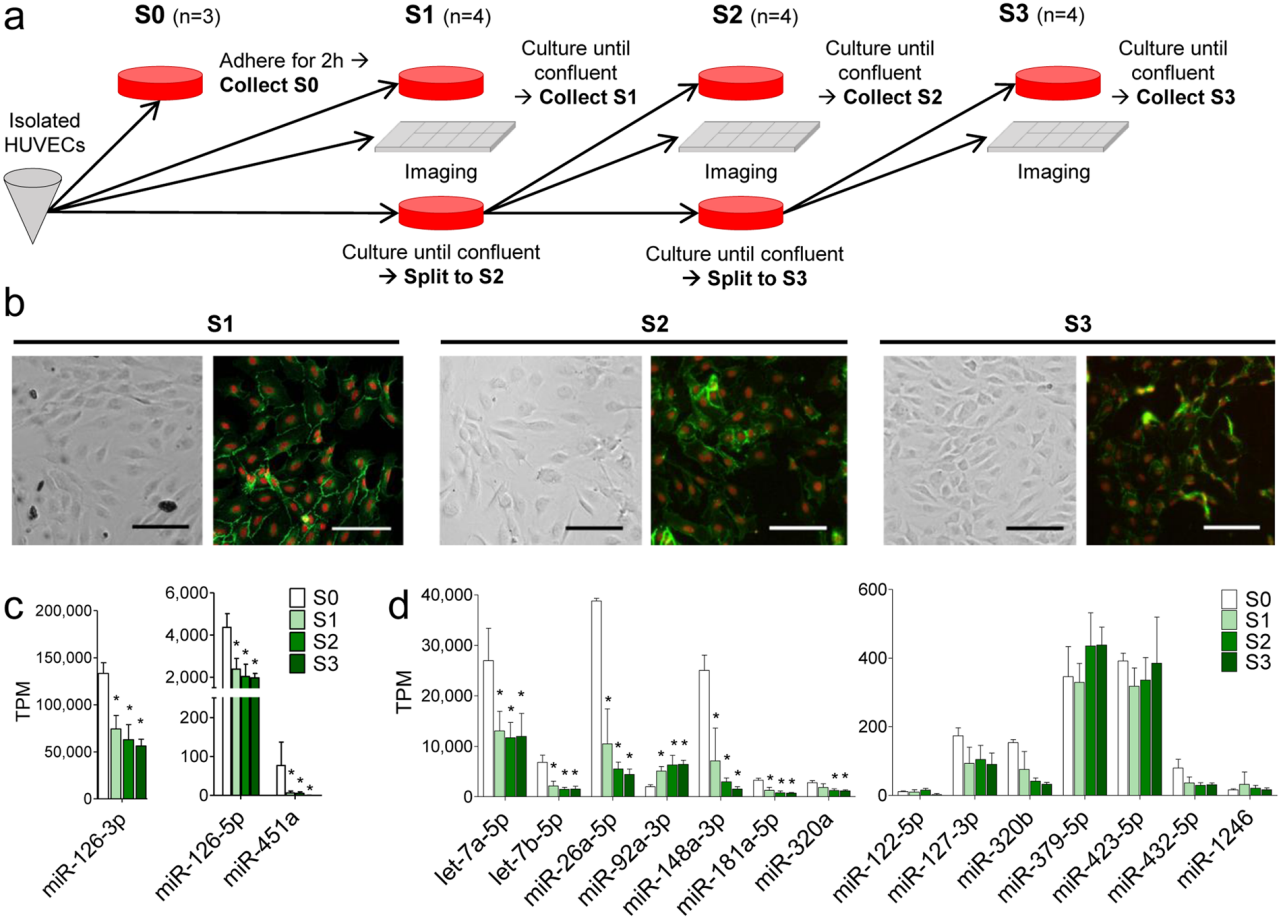
Cells isolated from umbilical cords are endothelial cells. (**a**) Sample collection outline. Endothelial cells isolated from one umbilical cord were split to four different vessels: (1) for S0 collection, (2) for S1 collection, (3) for subculturing and (4) for imaging. S0 had three and S1, S2 and S3 four biological replicates originating from four independent donors. (**b**) Cultured cells were visualized with endothelial specific VE-cadherin staining (green). Nuclei were stained with Draq5 (orange). Scale bar = 100 µm. (**c**) The expression of endothelial specific miR-126-3p and miR-126-5p, and red blood cell specific miR-451a from samples S0-S3. (**d**) The numbers of FBS enriched miRNAs in samples S0–S3. Data is expressed as mean +/− SD (n = 3-4), and results are considered significant at p < 0.05 when compared to S0 (*). TPM = transcripts per million.

To gain information on endothelial miRNA profiles and to study the changes between tissue-derived endothelial cells and cultured cells, we performed a miRNA-sequencing experiment. The samples were collected at endothelial cell extraction from umbilical cords (S0) and from three subsequent cell passages (S1–S3) (Fig. 1a). S0 samples represent the tissue-derived endothelial cells, which have grown in the presence of flow, and S1 to S3 samples are adjusted to static cell culture conditions. Of note, in standard HUVEC extraction, all endothelial cells isolated from one umbilical cord (donor) are plated and grown to confluence. In this experiment, however, due to collection of several types of samples, only 29–36% of the harvested endothelial cells were plated for subsequent cell passages. Therefore, more population doublings were required from one passage to another than in standard culturing resulting in aged cells at S3 and cellular senescence by S6 (Supplementary Fig. S1).

To confirm that the cells isolated from umbilical cords were endothelial cells, we first identified vascular endothelial cadherin (VE-cadherin) from the cell cultures. VE-cadherin is an endothelial cell-specific cadherin located at intercellular junctions^12^. The isolated cells had morphology characteristic of endothelial cells, and the stained samples were VE-cadherin positive showing that the cultures contained only endothelial cells (Fig. 1b). Also, the level of red blood cell enriched miR-451a^13^ was found to be significantly lower than the endothelial-enriched miRNAs miR-126 and miR-126-5p^14, 15^, indicating an absence of red blood cell contamination (Fig. 1c). In addition, we investigated the fetal bovine serum (FBS) enriched miRNAs from the data to see whether FBS supplements in S1–S3 cultures would affect the endothelial miRNA profile significantly. However, out of 14 miRNAs only one FBS-enriched miRNA, miR-92a, responded accordingly, but miR-92a has been previously shown to be subdued by high-shear environment^16^, thus move from flow to static environment would be expected to increase its expression (Fig. 1d). These data show that the samples used for miRNA-profiling were pure endothelial cell populations.

**Figure 2:**
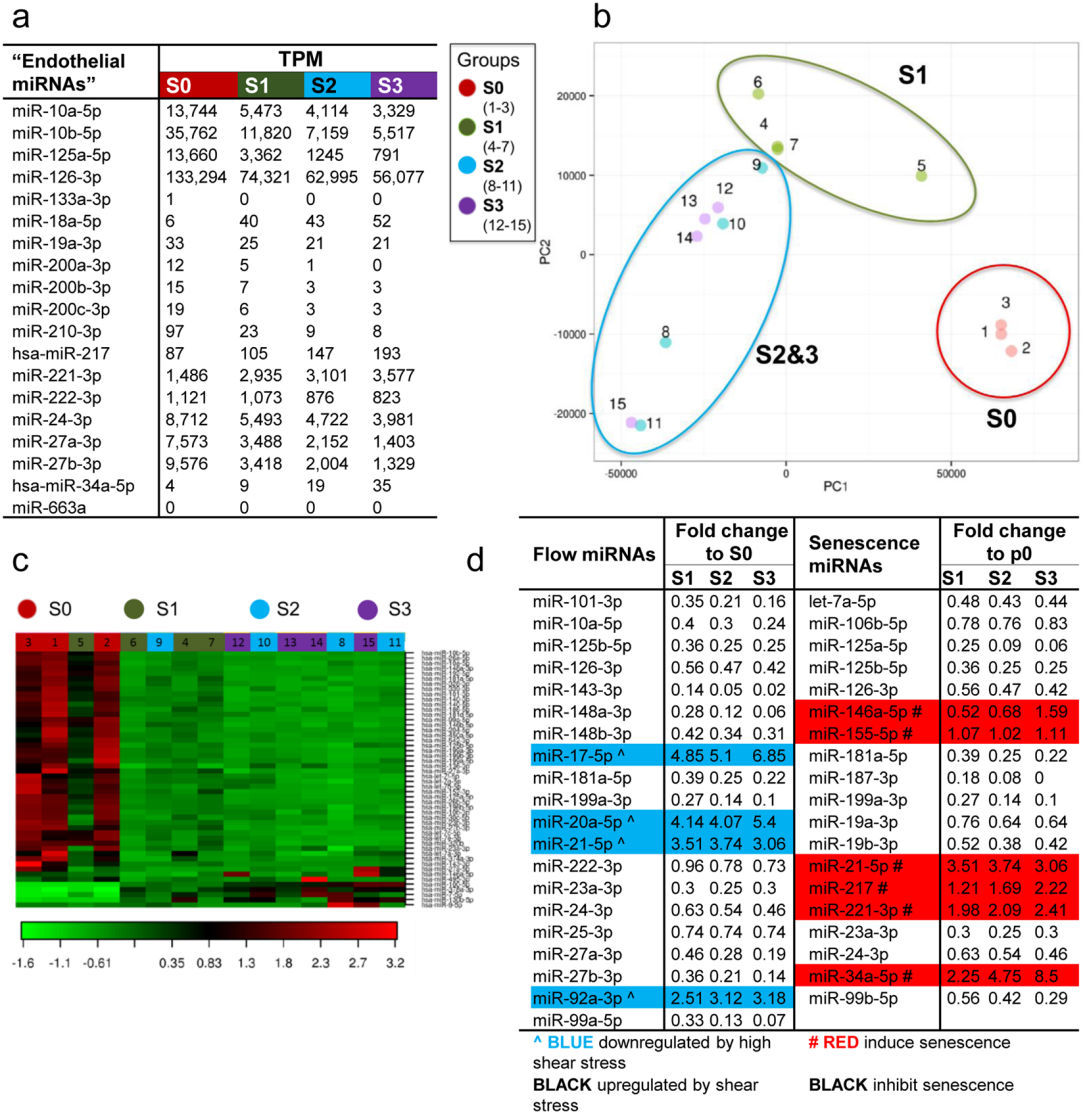
miRNA profile changes when cells move from tissue environment to cell culture. (**a**) miRNAs associated with endothelial cells (18–23). (**b**,**c**) Principal Components Analysis (**b**) and heat map and unsupervised hierarchical clustering by samples and miRNAs (**c**) were conducted on the 50 miRNAs with the highest coefficient of variation based on normalized counts across all samples. (**d**) Flow and senescence associated miRNAs (24–33). TPM = transcripts per million.

In the literature, miRNAs found from endothelial cells or miRNAs that have a functional effect in them are often referred to as endothelial miRNAs or “endomiRNAs”^17–22^. In the data presented here, many of these miRNAs (e.g. miR-10a/b, miR-24/27, miR-125a, miR-126 and miR-221/222) are abundant, while others are present in relatively low amounts (e.g. miR-18a, miR-19a, miR-34a, miR-200a/b/c, miR-210 and miR-217), or not at all (e.g. miR-133a and miR-663) (Fig. 2a). For example, the amount of miR-210 is about 13 times higher in tissue-derived endothelial cells (S0) than in cultured endothelial cells (S2-3), but the overall expression is low (Fig. 2a and Supplementary Table S1). Much alike, the expression of senescence-associated miRNAs miR-217 and miR-34a increases in aging cells compared to young cells by 2.2- and 8.5-fold, respectively, but the actual numbers of the miRNAs are low (Fig. 2a and Supplementary Table S1).

To characterize the differences between sample groups, Principal Components Analysis (PCA), unsupervised hierarchical clustering and heat map analyses on the top 50 microRNAs with largest variation across all samples were performed. PCA separated S0 samples from other groups and showed a shift from S1 samples to S2/S3 samples (Fig. 2b). Further analysis with heat map and two-way hierarchical clustering showed two distinct signatures, one for S0 and the other for S1–S3 (Fig. 2c). Again, a shift from S1 to S3 samples was seen. For most signature miRNAs, the relative expression was higher in S0 cells compared to S1–S3. Overall, several flow-responsive miRNAs were found to be downregulated together with senescence-inhibiting miRNAs in S1–3 compared to S0, whereas miRNAs associated with senescence-induction and static environment were upregulated, thus illustrating the change from tissue environment to static cell culture, and indicating cellular aging (Fig. 2d)^11, 16, 23–30^.

### From Tissue to Cell Culture: Most Abundant miRNAs and miRNA Families

**Figure 3:**
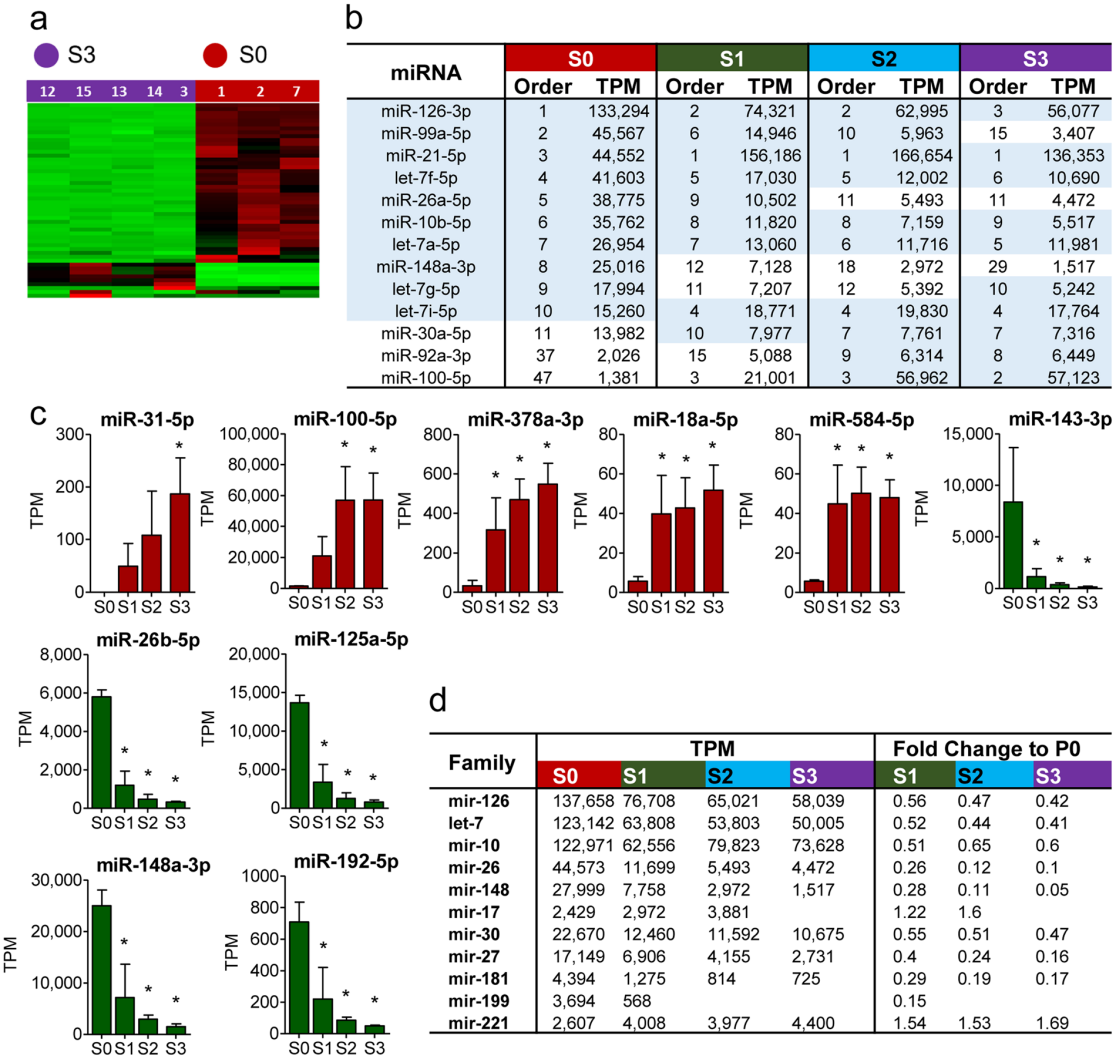
Most abundant miRNAs and miRNA Families. (**a**) Heat map showing unsupervised hierarchical clustering of S0 and S3 samples based on the 50 miRNAs with the highest coefficient of variation based on normalized counts across all samples. (**b**) Ten most abundant miRNAs in S0, S1, S2 and S3 samples. (**c**) Five most upregulated and downregulated miRNAs when S0 is compared to S1–S3. (**d**) Ten most abundant miRNA families among 50 most abundant miRNAs. Data is expressed as mean +/− SD (n = 3-4), and results are considered significant at p < 0.05 when compared to S0 (*). TPM = transcripts per million.

Most abundant miRNAs. Tissue-derived freshly isolated endothelial cells (S0) had clearly distinct profile from cultured cells (S1–S3), and the difference was most drastic when comparing S0 cells to S3 (Fig. 3a). The overall miRNA content was significantly higher in S0 compared to S1–S3. When comparing the total amount of the top 50 miRNAs in all four samples, the percentages for S1, S2, and S3 were 73%, 71% and 63%, respectively, of the total S0 miRNA quantity. The most downregulated miRNA was miR-126 and the most upregulated miR-21 (Table S1). Ten most abundant miRNAs, likely to be essential for endothelial cell function are shown in Fig. 3b. The most abundant miRNA in S0 was miR-126, whereas in S1–S3, it was miR-21, and by S3, miR-126 had dropped down to the third place, after miR-21 and miR-100. miR-148a, miR-99a and miR-26a were abundant in S0, but no longer in top 10 miRNAs in S3 where they were replaced by miR-100, miR-30a and miR-92a. When considering fold changes, the most upregulated miRNAs were miR-31, miR-100, miR-378a, miR-18a, and miR-584, whereas the most downregulated ones were miR-143, miR-26b, miR-125a, miR-148a, and miR-192 (Fig. 3c). The full expression data (TPMs, counts and all comparisons for differential expression) is available in Supplementary Table S1).

Families and Clusters. miRNA families consist of miRNAs having almost identical mature sequences and therefore both common and unique targets, whereas cluster miRNAs are transcribed from the same genomic location. The three most abundant miRNA families among 50 most abundant miRNAs in the miRNA-seq data were mir-126, let-7 and mir-10, and accordingly, the three most prevalent genomic locations were mir-126 and clusters of let-7a-1/7f-1/7d, and miR-99b/let-7e/miR-125a (Fig. 3d, Supplementary Table S1). Overall, the family abundance predominantly decreased from S0 to S3 for all, except for mir-221 and mir-17. mir-221 family consists of miR-221 and miR-222. Although miR-221 and miR-222 arise from the same genomic location, the quantity of miR-222 decreases steadily whereas that of miR-221 increases 2.4-fold from S0 to S3. mir-17 family does not appear in the top 50 miRNAs in S0, but two miRNAs, namely miR-20a and miR-17, climb to top 50 by S1, and miR-93 by S2. miR-20a and miR-17 belong to miR-17–92 cluster and miR-93 is from their paralogous miR-106b-25 cluster. The full table of families and clusters is available in (Supplementary Table S1).

### From young to old: Gene Ontology Enrichment Analysis

**Figure 4:**
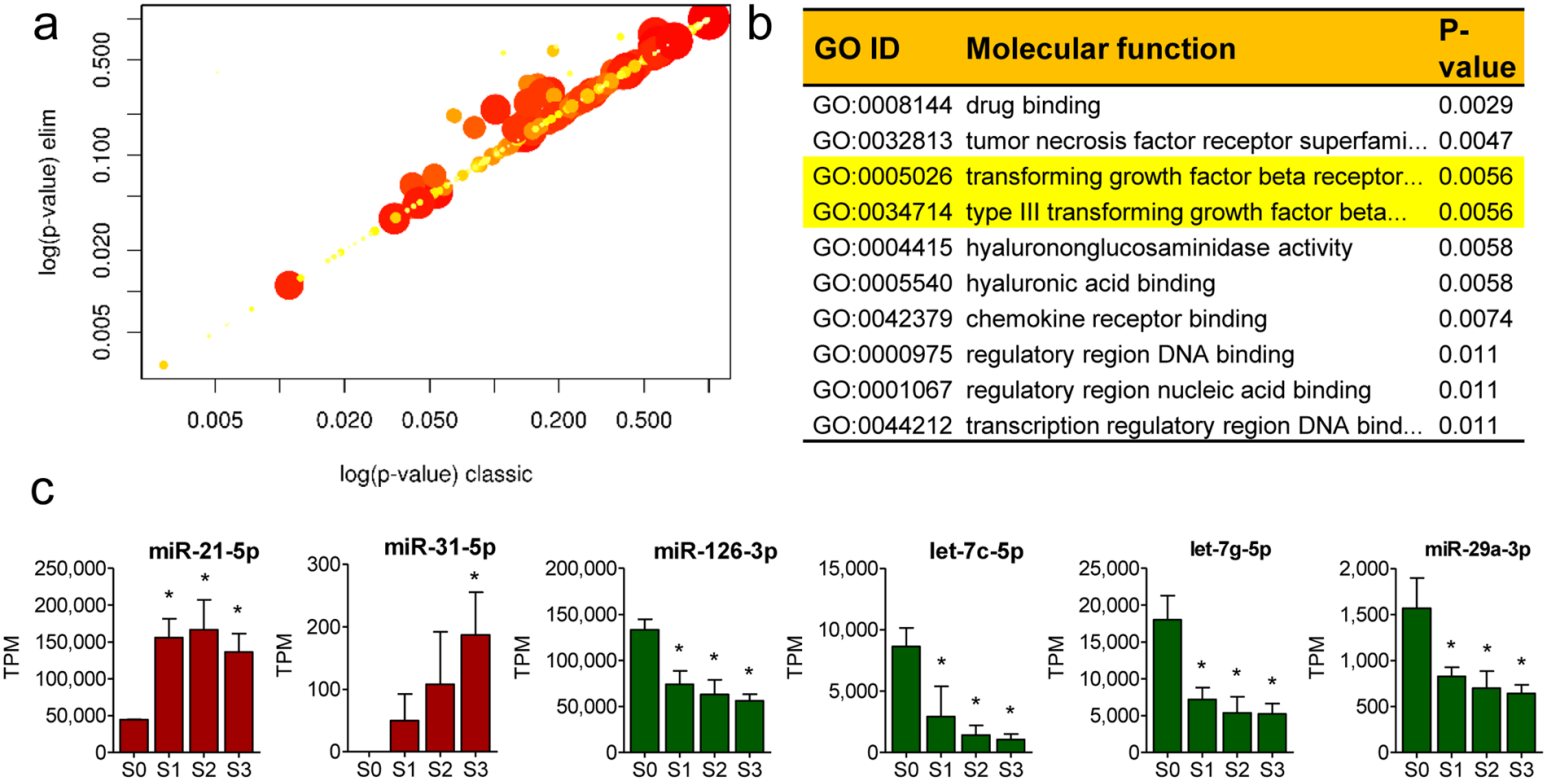
Gene Ontology Enrichment Analysis. (**a**) Scatter plot for significantly enriched GO terms (Molecular function) predicted to be associated with differentially regulated experimentally validated miRNA targets. Plot shows a comparison of the results obtained by two statistical tests used. Values along diagonal are consistent between both methods with values in the bottom left of the plot corresponding to the terms with most reliable estimates from both methods. Size of dot is proportional to number of genes mapping to that GO term and colouring represents number of significantly differentially expressed genes corresponding to the term with dark red representing more terms and yellow representing fewer. (**b**) Significant GO terms for the target genes predicted of being differentially expressed between S0 and S1, and their corresponding annotations for molecular functions. Data is expressed as mean +/− SD (n = 3-4), and results are considered significant at p < 0.05 when compared to S0 (*). TPM = transcripts per million.

To gain insight into the biological functions of the different miRNA networks in tissue-derived and cultured cells, Gene Ontology (GO) Enrichment Analysis was performed on the samples. Two different statistical tests were used and the results compared to gain the most relevant GO terms for affected molecular functions. The majority of the overrepresented terms were not statistically significant (Fig. 4a). However, among the top four molecular functions that were statistically significant, were two hits to transforming growth factor beta (TGF-ß) signalling pathway (Fig. 4b and Supplementary Fig. S2), which plays an essential role in mediating cell proliferation, migration, apoptosis and differentiation^31^. It has also been implicated in the development of tissue fibrosis and phenotypic conversion of several cell types, including endothelial cells, to activated mesenchymal cell types^32, 33^. The molecular mechanisms and intracellular cascades that lead to endothelial to mesenchymal transition have not been thoroughly elucidated, but the involvement of miRNAs in the process has been previously published^34–38^. In the data, pro-fibrotic miRNAs, miR-21-5p and miR-31-5p were upregulated and anti-fibrotic miRNAs, miR-126-3p, and the members of the let-7 and mir-29 families downregulated in S1–S3 cells compared to S0 (Fig. 4c), suggesting a transition towards mesenchymal cell type in aging cells.

### Putative miRNAs and IsomiRs

#### Putative miRNAs

As many miRNAs may remain undiscovered, we determined the putative novel miRNAs (put-miRs) in the data, and the changes in their expression across the samples. Put-miRs were identified from the sequences that did not map to any organism found in miRBase, or to other known RNA sequences, and their changes across sample groups were observed. The cut-off for detection was set to 30 TPM (transcripts per million). In contrast to most miRNAs in the data, the amounts of the two identified put-miRs increased from S0 to S1–S3 (Table 1), but their overall amounts were very low. Put-miR-1 resides on chromosome 10 between genes *rhotekin* 2 (*RTKN2*) and *zinc finger protein* 365 (*ZNF36*5). It is predicted of being repressed or low activity region in HUVECs according to multivariate genome-segmentation ENCODE data^39, 40^ (data not shown), whereas put-miR-2 resides on weak enhancer region on open chromatin in the first intron of *potassium channel tetramerization domain containing 5* (*KCTD5*) on chromosome 16.

**Table 1:** Putative miRNAs. Putative novel miRNAs were predicted from sequences, which did not map to any organism in miRBase or to other known RNA sequences. TPM = transcripts per million.

Identifier	Chr	Strand	Start	Stop	Sequence	TPM				Fold change		
						S0	S1	S2	S3	S1	S2	S3
put-miR-1	10	+	64,075,172	64,075,190	GAGTGTGAGTCTGAAACTG	1.3	41.3	114.5	79.5	30.9	85.9	59.6
put-miR-2	16	+	2,737,223	2,737,253	CTCTGGTGATGAAATGGAACGTTTCTGATGG	0	30.8	30.5	33.3	—	—	—

#### IsomiRs

**Figure 5:**
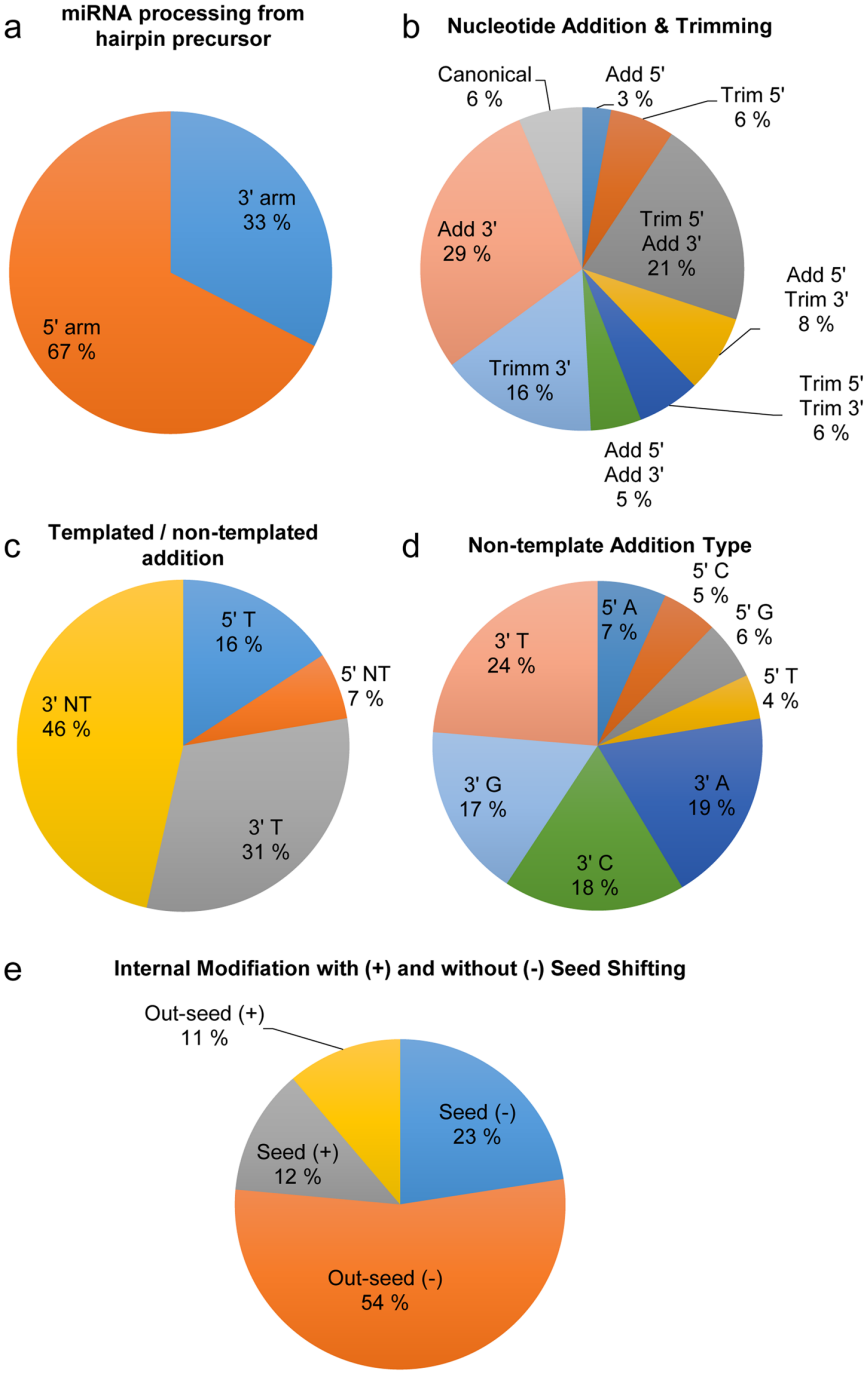
IsomiRs. (**a**) Percentages for mature miRNAs that are processed from the 5′ or 3′ arms of the hairpin precursor. The numbers are based on unique tags (n = 15). Results for group-wise comparisons were similar to the shown diagram. (**b**) Percentages for nucleotide addition and/or trimming occurring at the 5′ and 3′ ends of miRNAs based on counted unique tags (n = 15). No deviations were seen in group-wise comparisons. (**c**) Percentages for templated and non-templated nucleotide additions at 5′ and 3′ ends of the miRNAs are shown (n = 15). The numbers are based on identified unique tags. The results for group-wise comparisons were identical to the diagram shown here. (**d**) Percentages for non-templated addition types to 5′ and 3′ ends of the miRNAs are shown (n = 15). The numbers are based on unique tags, and group-wise comparisons resulted in identical diagrams as shown here. (**e**) Percentages based on unique tags for internal modifications in the seed region or outside seed region resulting in seed shifting (+) and without seed shifting (−) are shown (n = 15). The group-wise results were identical to the diagram shown here.

The miRNA isomers were extracted from the sequencing data with an online tool called DeAnnIso^41^. The sample wise results are available in Supplementary Table S1, and the full DeAnnIso analyses can be accessed through the sample identifiers provided in the table. Combined results for miRNA processing are shown in Fig. 5. Analysis on miRNA processing from the hairpin precursors showed a clear preference for 5′ arm selection across all samples (Fig. 5a). The results for individual and grouped samples were similar with a slight decrease and increase seen for respective 3′ and 5′ arm percentages from S0 to S3. Nucleotide addition to 3′ end of the miRNA, 3′ addition combined with 5′ trimming, and 3′ trimming of the miRNA were the most common modifications detected across all samples and the sample- or group-wise results showed no deviation from the results (Fig. 5b). Of the additions, majority of 5′ additions were of the template type, whereas for 3′ additions the major type was the non-templated addition (Fig. 5c). Closer inspection of the non-templated additions showed a slight preference for addition of T to the 3′ end, whereas for 5′ end, addition of A was slightly more popular than the additions of the other nucleotides (Fig. 5d). Internal modifications without seed shifting were more abundant than the modifications causing seed shifting (Fig. 5e). The majority of the modifications occurred outside seed region in miRNAs without seed shifting, but the percentages were closer to being equal in modifications causing a seed shift.

## Discussion

Cell culturing of primary endothelial cells derived from umbilical cords was first reported by Jaffe *et al*.^42^. Since then, advances in vascular endothelial cell biology have greatly enhanced the knowledge on their role in pathophysiological conditions ranging from cardiovascular disease to cancer and inflammation^43^. Endothelial cell diversity is apparent from both gene expression and miRNA profiling of different sites in the vascular endothelium, and specific arterial and venous profiles have been identified^44–46^. Nevertheless, the understanding of the endothelial cell biology has vastly benefited from the studies employing cultured human umbilical vein endothelial cells (HUVECs) and the studies have led to many groundbreaking revelations in the field, such as the identification of the endothelium-derived relaxing factor nitric oxide NO^47^. The status of HUVECs among the most popular endothelial models is mostly due to the readily available human tissue source (umbilical cords) and to the fact that the endothelial cell yield is relatively high and the isolated cell population is pure. The isolation protocol produces confluent primary endothelial cells within a week and the cells can be reliably subcultured for 4-5 passages^43^. To our knowledge, the data presented in this study is the first large-scale miRNA profiling using miRNA-sequencing that compares freshly isolated and cultured HUVECs. The profiling revealed major alterations in endothelial miRNA signatures between tissue-derived, cultured and aging endothelial cells.

The first study to profile endothelial miRNAs was executed in young HUVECs using microarray technology^48^. 17 out of the 27 miRNAs listed as highly expressed in the study were also present in our data among the 50 most abundant miRNAs, although not necessary among the highest-ranking ones. In the past profiling studies of endothelial cells^49^, the two most frequently encountered miRNAs have been miR-126 and miR-21, which were also the most abundant miRNAs in this study. In general, most of the miRNAs that were associated with endothelial function in previous studies were also detected here^17–22^. However, some of the previously detected endothelial miRNAs were present in very low numbers or not at all. As endothelial cells are very plastic in nature, and they can alter their phenotype according to the surrounding vascular microenvironment, the observed differences in the miRNA expression between the previous studies and the present study can arise from the different cellular states and environmental cues the cells were exposed to. Furthermore, the significance level for miRNA expression for biologically meaningful effects has not been determined, i.e. even small miRNA amounts might be of importance. However, for the establishment of biologically meaningful miRNA-target interactions, it is crucial to ensure that the effects of miRNAs are investigated in appropriate cell types, as miRNA overexpressed in any, also inappropriate, cell type, will find targets to regulate and therefore alter the cell function^6^.

Of note, the most considerably downregulated miRNA in the cell culture cells compared to the tissue-derived cells was miR-143. Endothelial cells are known to participate in extracellular miRNA trafficking by both secreting and receiving miRNA cargos^50^. One of the earliest studies of exosomal miRNA transfer demonstrated the transport of functional miR-143 and its cluster member miR-145 from endothelial cells to smooth muscle cells^24^, but the study has been criticized because of the higher expression of the cluster members in smooth muscle cells compared to endothelial cells^50^. More recently, another study demonstrated the transfer of the same miRNAs to opposite direction, from smooth muscle cells to endothelial cells^51^. Here, miR-143 was found to be among the 20 most abundant miRNAs in tissue-derived (S0) cells suggesting its prominent presence *in situ*, whereas miR-145 was hardly detected. Though the overall number of miRNAs dropped from freshly isolated (S0) to cultured cells (S1), and further in aging cells (S3) the expression of miR-143 dropped even more drastically: the expression was an order of magnitude lower in S1 and two orders of magnitude lower in S3 in comparison to S0. It cannot be concluded from the present data whether miR-143 in endothelial cells results from the intrinsic expression or from the transfer from smooth muscle cells, but along with the canonical form of the miRNA, a rich set of its isomiRs was detected, supporting its endothelial origin.

Data analysis revealed a striking difference between tissue-derived and cultured endothelial cells, logical when considering the high endothelial plasticity and the differences between the tissue and cell culture environment^7, 33, 52^. In the tissue environment, endothelial cells are subject to biomechanical forces caused by circulating blood and the flow characteristics, such as velocity and the flow patterns (laminar vs. turbulent), influence the cell function^53^. In addition, tissue cells rely on blood-derived oxygen and the switch to culture-environment exposes the cells to ambient air and higher oxygen tension, which leads to a state of relative hyperoxia compared to tissue environment^54^. Also, the lack of flexible extracellular matrix and interacting neighbouring cells in the cell culture environment alter the appearance and function of the cells^7, 55^. All these factors are likely to add up to the observed shift in the endothelial miRNA profile from tissue-derived cells to cultured cells, and to affect the overall miRNA content, arising from both intracellular expression and extracellular miRNA uptake.

In aging blood vessels, accumulation of senescent cells compromises the endothelial barrier integrity and function, and predisposes to aging-related diseases, such as cardiovascular disease, type 2 diabetes and cancer. Moreover, senescence spreads through paracrine signalling to neighbouring cells, which also become senescent^56, 57^. Senescent cells have unique gene expression patterns and miRNA signatures that set them aside from proliferating and quiescent cells, but are highly similar to the expression patterns in major age-related diseases^11, 58^. Two miRNAs that have been previously associated with inflammatory cell aging and cellular senescence are miR-21 and miR-126^58^. Accordingly, senescence-inducing miR-21 was upregulated and senescence-inhibiting miR-126 was downregulated in aging endothelial cells in our study. Curiously, these two miRNAs have also been associated with phenotypic transition of endothelial cells into mesenchymal cells (EndoMT)^34^, suggesting a link between endothelial aging and EndoMT. Importantly, senescence-associated secretory phenotype has been previously shown to turn senescent fibroblasts into proinflammatory cells that promote tumour progression by inducing epithelial-to-mesenchymal transtition (EMT) in nearby epithelial cells^59^. The principal inducer of both EndoMT and EMT is TGFß, which is also the central signalling pathway connecting inflammation, senescence and aging-related diseases^32, 34, 58^. Of note, GO term enrichment analysis on our data predicted the pathway of being differentially regulated between tissue-derived young cells and aging cultured cells, and the subsequent inspection of EndoMT-related miRNAs indicated a possible shift towards mesenchymal phenotype in aging cells.

High-throughput small RNA sequencing technology combined with advanced computational approaches has revealed that a single miRNA locus gives rise to complex and dynamic repertoire of miRNA isoforms, isomiRs, which differ in their length and sequence composition^41^. Although originally dismissed as sequencing errors, isomiRs have since been demonstrated of being constitutively produced in human cells and to differ between tissue types and states, as well as to depend on gender, race and disease subtype^60, 61^. Different isomiRs originating form the same miRNA precursor have been shown to target different genes and pathways, which extends the regulatory output of a given miRNA locus considerably^60^. In this study, we have catalogued the isomiRs extracted from the endothelial cells, and identified the modifications leading to their formation. Although 3′ modifications are more common form of variations, 5′ modifications were also detected, and are likely to affect mRNA targeting and lead to a broader range of miRNA action^60, 62^.

To summarize, the profiling of endothelial cells revealed distinct miRNA profiles between tissue-derived and cultured cells. Predictably, flow- and senescence-responsive miRNAs were seen to alter, but alterations were also detected in miRNAs participating in EndoMT indicating a spontaneous phenotypic transition of the cells due to cell culture environment and aging. The comprehensive miRNA profiling by miRNA sequencing provides both novel and unique information on endothelial miRNAs of tissue-derived and aging cultured cells and provides a rich catalogue for future studies.

## Materials and Methods

### Cell Culture and Sample Collection

Umbilical cords were obtained from the maternity ward of the Kuopio University Hospital. The collection, cell extraction and following experiments were approved by the Research Ethics Committee of the Hospital District of Northern Savo, Kuopio, Finland. Informed written consent was received from all participants and the experiments were performed in accordance with the relevant guidelines and regulations. Human Umbilical Vein Endothelial Cells (HUVECs) were extracted with collagenase (0.3 mg/ml) digestion immediately after the cord collection. The cells were cultivated in Endothelial Cell Basal Medium (Lonza) with recommended supplements (EGM SingleQuot Kit Supplements & Growth Factors, Lonza). At cell extraction, Sample 0 (S0) cells were adhered for 2 h on fibronectin-gelatin coated T25 flasks, and the samples were collected after rigorous washing. S1 samples were collected at the first cell passage, and S2 and S3 on the following two passages. For microRNA sequencing, cells of four donors were collected. On one donor, the cell yield was too low for S0 sample, and therefore the number of biological replicates is three for S0, and four for S1–S3.

### Endothelial cell imaging

HUVECs were fixed in 4% paraformaldehyde followed by pre-treatment with 0.2% Triton X-100 in PBS for 15 min. After washing with PBS and blocking with FBS for 15 min, cells were incubated with VE-cadherin antibody (Life Technologies, #A18353) for 2 h. The nuclei were stained with Draq5 (Thermo Scientific, #62251). Senescence β-Galactosidase Staining kit (Cell Signaling, #9860) was used according to the manufacturer’s protocol. Briefly, fixed cells were stained with β-Galactosidase Staining solution and sealed with parafilm for overnight incubation at 37 C. Cells were imaged using Zeiss Observer.Z1 microscope.

### MicroRNA Sequencing (miRNA-seq)

#### Library Preparation

RNA was extracted using miRCURY RNA isolation kit for cells and plants (Exiqon). miRNA profiling was conducted at Exiqon Services, Denmark. 300 ng of total RNA was converted into microRNA NGS libraries using NEBNEXT library generation kit (New England Biolabs) according to the manufacturer’s instructions. Each individual RNA sample had adaptors ligated to its 3′ and 5′ ends and converted into cDNA. cDNA was pre-amplified with specific primers containing sample specific indexes. After 15-cycle pre-PCR the libraries were purified on QiaQuick columns and the insert efficiency evaluated by Bioanalyzer 2100 instrument on high sensitivity DNA chip (Agilent Inc.). The microRNA cDNA libraries were size fractionated on a LabChip XT (Caliper) and a band representing adaptors and 15–40 bp insert excised using the manufacturer’s instructions. Samples were then quantified using qPCR and concentration standards. Based on quality of the inserts and the concentration measurements the libraries were pooled in equimolar concentrations. The library pools were finally quantified again with qPCR and optimal concentration of the library pool used to generate the clusters on the surface of a flowcell before sequencing using v3 sequencing methodology according to the manufacturer instructions (Illumina). Samples were sequenced on the Illumina NextSeq. 500 system.

#### Data Analysis

miRNA-seq data was mapped to miRBase (v21)^2^ and to genome version GRCh37 using Bowtie2 (2.2.2)^63^. The aligned reads were required to match the reference sequence perfectly and one mismatch was allowed in the first 32 bases of the read when mapping to the genome. No indels were allowed in the mapping. Novel miRNAs were predicted using mirPara^64^ and miRBase and isomiRs were detected using DeAnnIso with default settings defined in ref. 41. The cut-off for isomiRs listed in the Supplementary Table S1 was set to 100 counts. Putative novel microRNAs were predicted from the sequences that did not map to any organism found in miRBase, or to other known RNA sequences. Unsupervised analyses (Heatmaps, hierarchical clustering and Principal Components Analysis) were performed using R. The differential expression analysis was performed using the EdgeR statistical software package^65, 66^. For normalisation, the trimmed mean of M-values method based on log-fold and absolute gene-wise changes in expression levels between samples (TMM normalization) was used. GO analyses are done with R package TopGO^67^ with experimentally verified miRNA targets as input.

#### Data availability

miRNA-seq data has been deposited in NCBI’s Gene Expression Omnibus^68^ and are accessible through GEO Series accession number GSE94410 (http://www.ncbi.nlm.nih.gov/geo/query/acc.cgi?acc =GSE94410). DeAnnIso analyses can be accessed using the sample identifiers provided in the Supplementary Table S1.

## Electronic supplementary material

Supplementary Material

Supplementary Table S1
