# Supplementary material for: MicroRNA Profiling Reveals Distinct Profiles for Tissue-Derived and Cultured Endothelial Cells
Source: Sci Rep. 2017 Sep 8;7:10943. doi: 10.1038/s41598-017-11487-4 (PMC5591252; doi:10.1038/s41598-017-11487-4)
Supplement: Supplementary file 1 — Supplementary Material [file 41598_2017_11487_MOESM1_ESM.pdf]

MicroRNA Profiling Reveals Distinct Profiles for Tissue-Derived and Cultured Endothelial Cells

Suvi M. Kuosmanen, Emilia Kansanen, Virve Sihvola and Anna-Liisa Levonen

SUPPLEMENTARY INFORMATION

SUPPLEMENTARY TABLE LEGENDS

Supplementary Table S1. miRNA-seq Data. Table includes miRNA counts, normalized values (TPM), differential expression analyses for all sample groups, miRNA clusters and families and isomiRs.

SUPPLEMENTARY FIGURE LEGENDS

Supplementary Figure S1. Senescence staining of standard HUVEC extraction and miRNA sequencing sample collection. A-D. Passages 4, 8, 12 and 16 from standard HUVEC extraction. **E-F.** S3 and S6 from miRNA sequencing sample collection. Scale bar = 100 µm

Supplementary Figure S2. GO network generated from the GO terms predicted of being enriched for molecular functions. Nodes are coloured from red to yellow with the node with the strongest support coloured red and nodes with no significant enrichment coloured yellow. The five nodes with the strongest support are marked with rectangular nodes.

SUPPLEMENTARY FIGURES

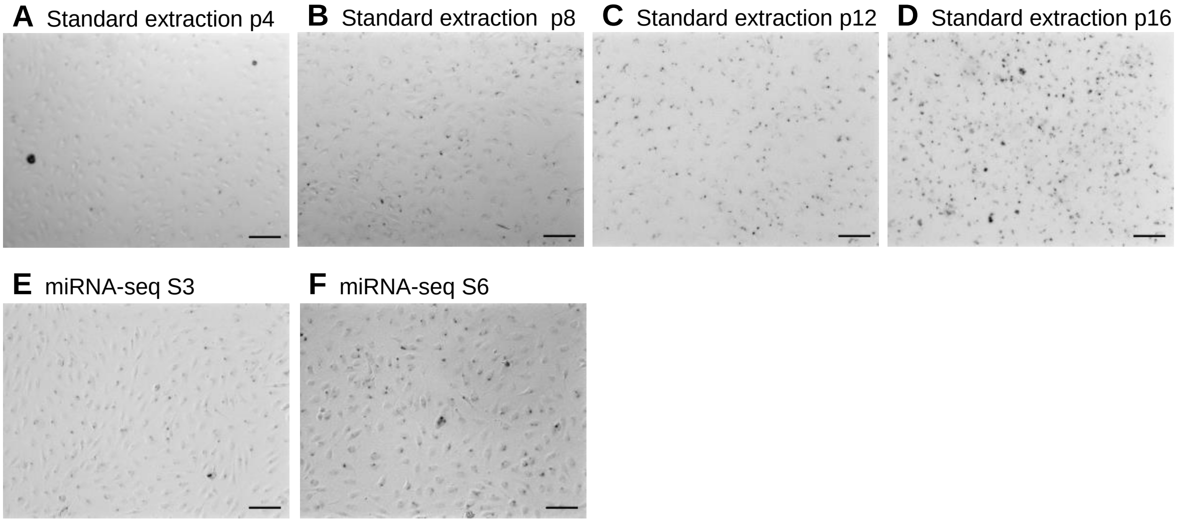

Supplementary Figure S1.

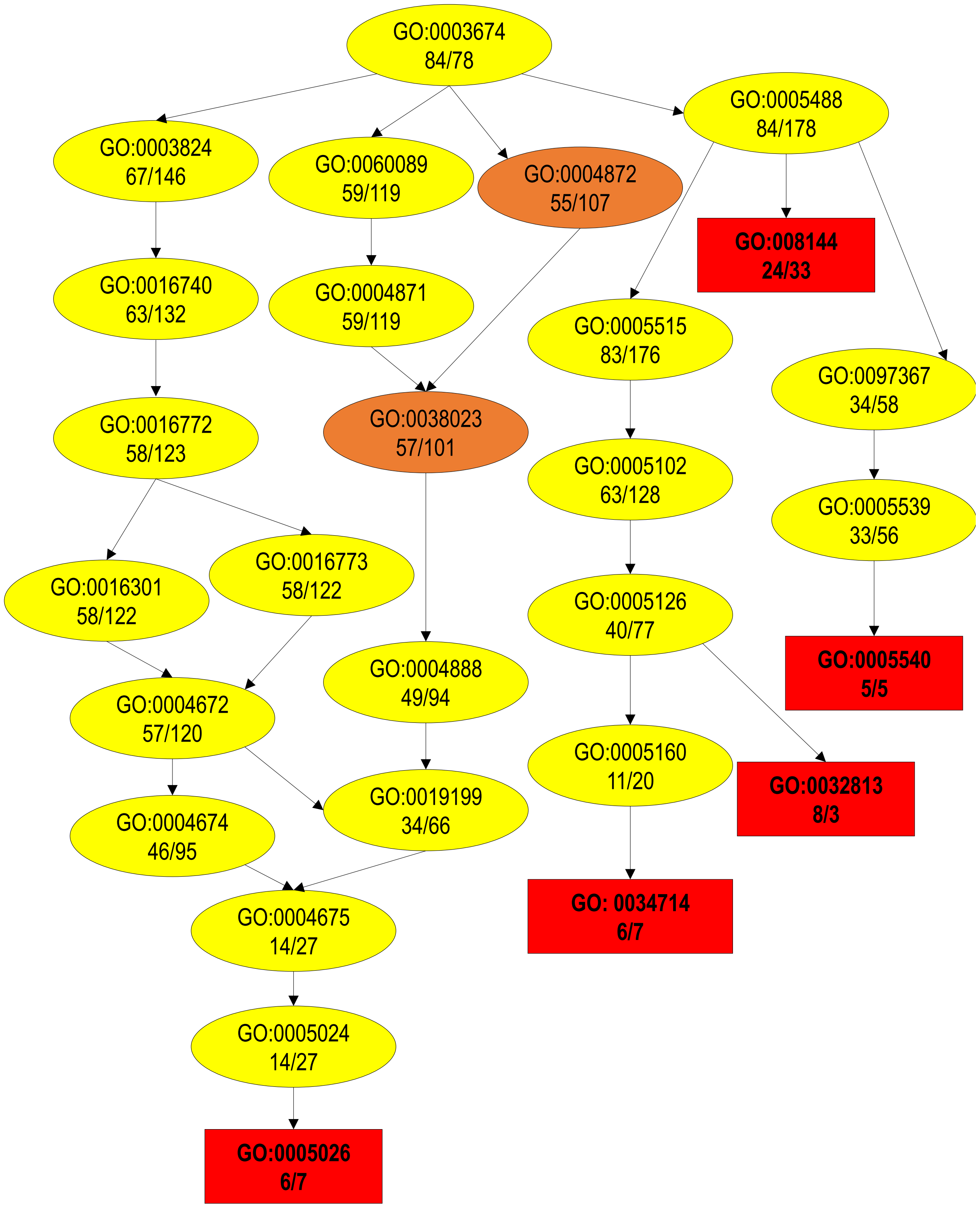

Supplementary Figure S2.
